# Oleic Acid and Eicosapentaenoic Acid Reverse Palmitic Acid-induced Insulin Resistance in Human HepG2 Cells via the Reactive Oxygen Species/*JUN* Pathway

**DOI:** 10.1016/j.gpb.2019.06.005

**Published:** 2021-02-23

**Authors:** Yaping Sun, Jifeng Wang, Xiaojing Guo, Nali Zhu, Lili Niu, Xiang Ding, Zhensheng Xie, Xiulan Chen, Fuquan Yang

**Affiliations:** 1 Key Laboratory of Protein and Peptide Pharmaceuticals & Laboratory of Proteomics, Institute of Biophysics, Chinese Academy of Sciences, Beijing 100101, China; 2 University of Chinese Academy of Sciences, Beijing 100049, China

**Keywords:** Free fatty acid, Insulin resistance, Quantitative proteomics, Calcium, ATP

## Abstract

Oleic acid (OA), a monounsaturated fatty acid (MUFA), has previously been shown to reverse saturated fatty acid palmitic acid (PA)-induced hepatic **insulin resistance** (IR). However, its underlying molecular mechanism is unclear. In addition, previous studies have shown that eicosapentaenoic acid (EPA), a ω-3 polyunsaturated fatty acid (PUFA), reverses PA-induced muscle IR, but whether EPA plays the same role in hepatic IR and its possible mechanism involved need to be further clarified. Here, we confirmed that EPA reversed PA-induced IR in HepG2 cells and compared the proteomic changes in HepG2 cells after treatment with different **free fatty acids** (FFAs). A total of 234 proteins were determined to be differentially expressed after PA+OA treatment. Their functions were mainly related to responses to stress and endogenous stimuli, lipid metabolic process, and protein binding. For PA+EPA treatment, the PA-induced expression changes of 1326 proteins could be reversed by EPA, 415 of which were mitochondrial proteins, with most of the functional proteins involved in oxidative phosphorylation (OXPHOS) and tricarboxylic acid (TCA) cycle. Mechanistic studies revealed that the protein encoded by *JUN* and reactive oxygen species (ROS) play a role in OA- and EPA-reversed PA-induced IR, respectively. EPA and OA alleviated PA-induced abnormal adenosine triphosphate (**ATP**) production, ROS generation, and **calcium** (Ca^2+^) content. Importantly, H_2_O_2_-activated production of ROS increased the protein expression of *JUN*, further resulting in IR in HepG2 cells. Taken together, we demonstrate that ROS/*JUN* is a common response pathway employed by HepG2 cells toward FFA-regulated IR.

## Introduction

Insulin resistance (IR) refers to a process where insulin action is impaired in insulin-targeted tissues, such as skeletal muscle, liver, and adipocytes. Previous studies have shown that IR first occurs in the liver, followed by skeletal muscle and adipose tissues [1]. When IR occurs, the liver fails to produce hepatic glycogen and inhibit gluconeogenesis, which further causes high blood glucose [2]. Additionally, hepatic IR also causes an abnormal accumulation of lipids and alterations in lipid synthesis [3]. The dual anomalies of glucose level and lipid content often result in chronic liver diseases, including nonalcoholic fatty liver, nonalcoholic steatohepatitis, and even hepatic cirrhosis [4].

Currently, the association between free fatty acids (FFAs) and IR is widely recognized [5]. Saturated fatty acids (SFAs) and monounsaturated fatty acids (MUFAs) have opposite effects on hepatic IR [6]. Palmitic acid (PA), the most prevalent circulating SFA, has been reported to cause hepatic IR through the abnormal accumulation of ceramides, diacylglycerols, and triglycerides [7], [8] or through the impairment of cellular signaling pathways, typified by mitochondrial dysfunction, endoplasmic reticulum (ER) stress, and apoptosis [8]. Oleic acid (OA), the most common MUFA in the human diet, has been shown to reverse IR in primary hepatocytes by inhibiting the phosphorylation of the ribosomal protein S6 kinase 1 (S6K1) [9]. Despite all of these studies, no systematic analysis of the effects of SFAs and MUFAs on hepatocytes has been performed to date.

Previous research has also shown that eicosapentaenoic acid (EPA), a typical ω-3 polyunsaturated fatty acid (PUFA), could reverse SFA-induced IR through decreasing inflammation in skeletal muscle [10] or through regulating lipid metabolism in adipose tissues [11]. However, the questions of whether EPA can reverse hepatic IR and how EPA functions in the liver remain elusive. Additionally, if EPA and OA exert similar effects on PA-induced IR in hepatocytes, whether they utilize the same molecular mechanism also requires further investigation.

Due to the fact that HepG2 cells can normally respond to insulin and display reduced insulin signaling due to lipid accumulation, many researchers have used them to investigate the insulin signaling pathway and IR [12], [13]. Therefore, we also used HepG2 cells to build two cell models of hepatic IR to explore the effects of different FFAs on hepatocyte IR. Additionally, given that the stable isotope labeling with amino acids in cell culture (SILAC)-based quantitative proteomic method has good quantitative accuracy and reproducibility compared with other mass spectrometry (MS)-based quantitative methods, such as label-free or chemical labeling methods [14], SILAC was employed to elucidate cellular responses to various FFAs. Further biochemical experiments demonstrated that the reactive oxygen species (ROS)/*JUN* pathway was a common pathway utilized by OA and EPA to reverse PA-induced IR in HepG2 cells. Targeted exploration of this pathway may be useful in the treatment of metabolic diseases.

## Results

### Effects of PA, OA, and EPA on hepatic IR

**Figure 1:**
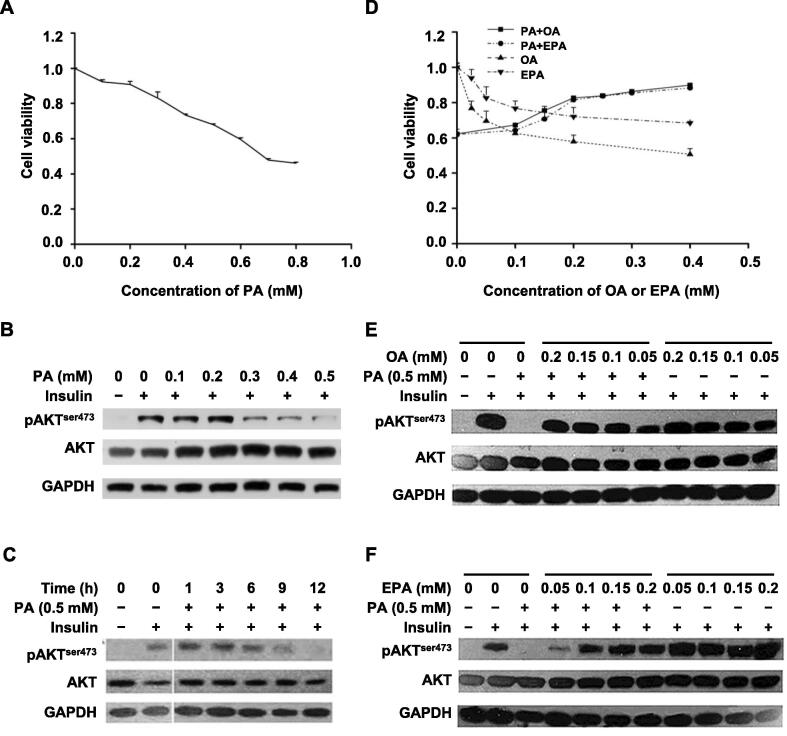
**Effects of PA, OA, and EPA on cell viability and IR in HepG2 cells** **A.** PA-induced inhibition of cell viability in HepG2 cells. HepG2 cells were treated with different concentrations of PA (0**–**0.8 mM) for 12 h. The cell viability was tested by MTT assay. Data were represented as mean ± SD. **B.** and **C.** PA-reduced insulin sensitivity in a concentration-dependent (B) and time-dependent (C) manner. HepG2 cells were treated with different concentrations of PA (0**–**0.5 mM) for 12 h (B) or with 0.5 mM PA for different time durations (0**–**12 h) (C), and then treated with 100 nM insulin for 20 min at 37 °C. Western blots of total lysates were performed using antibodies for indicated proteins. GAPDH was used as a loading control. **D.** Effects of OA and EPA on PA-induced inhibition of cell viability in HepG2 cells. HepG2 cells were incubated in the absence or presence of 0.5 mM PA with different concentrations (0**–**0.4 mM) of OA or EPA for 12 h. Data were represented as mean ± SD. **E.** and **F.** OA (E) and EPA (F) prevented PA-induced impairment of insulin signaling in a concentration-dependent manner. HepG2 cells were incubated in the presence or absence of 0.5 mM PA with (0.05**–**0.2 mM) or without OA (E) or EPA (F) for 12 h before insulin treatment (100 nM for 20 min at 37 °C). Western blots of total lysates were performed using antibodies for indicated proteins. PA, palmitic acid; OA, oleic acid; EPA, eicosapentaenoic acid; IR, insulin resistance; GAPDH, glyceraldehyde-3-phosphate dehydrogenase.

To elucidate the influence of PA on the hepatic insulin signaling pathway, we used a combination of 3-(4,5-dimethylthiazol-2-yl)-2,5-diphenyltetrazolium bromide (MTT) assays, dose–response experiments, and time-course experiments in HepG2 cells. First, PA displayed obvious growth-inhibitory effects on HepG2 cells in MTT assays (Figure 1A). The half maximal inhibitory concentration (IC50) of PA treatment in HepG2 cells was 0.65 mM. Therefore, 0.5 mM PA was chosen as the highest concentration for further experiments. Then, in dose–response and time-course experiments, the level of insulin-stimulated AKT phosphorylation (pAKT), which represents the activation level of insulin signaling [15], decreased with increasing concentrations of PA and increasing time of PA treatment (Figure 1B and C). These results indicated that PA could induce IR in HepG2 cells in a concentration- and time-dependent manner. As shown in Figure 1B and C, 0.5 mM PA treatment for 12 h was an optimal condition for a cell model of hepatic IR in HepG2 cells.

To investigate the effects of OA and EPA on PA-induced IR in HepG2 cells, MTT assays and dose–response experiments were performed. The MTT assay results showed that OA or EPA treatment alone inhibited cell viability, similarly to PA treatment, but combination treatment with PA and OA (PA+OA) or PA and EPA (PA+EPA) improved cell viability of HepG2 cells compared with PA treatment alone (Figure 1D). The growth-proliferation effect of PA+OA treatment was consistent with the previous observation that OA attenuated PA-induced apoptosis through OA-activated autophagy [16].

To explore whether OA or EPA could affect the cellular uptake of PA to alleviate PA-mediated cell death, we measured the content of PA in HepG2 cells after treatment with different FFAs by gas chromatography–tandem mass spectrometry (GC–MS/MS) using the external standard method. As shown in Figure S1A, OA or EPA alone did not affect the cellular uptake of PA. The treatment of PA+OA or PA+EPA increased the content of PA in HepG2 cells compared with PA treatment by itself. This result was consistent with a previous report that OA could produce PA by shortening chains in fatty acid cycling [17].

In addition to improving cell viability, both OA and EPA also attenuated PA-induced IR in HepG2 cells in a concentration-dependent manner (Figure 1E and F). Moreover, the reversal effects of OA and EPA on IR in hepatocytes were also observed in normal human L02 hepatocytes (Figure S1B). Therefore, 0.5 mM PA, 0.2 mM OA, and 0.15 mM EPA were chosen for further quantitative proteomic analyses.

### Quantitative proteomic results of OA- or EPA-mediated reversal of PA-induced IR in HepG2 cells

To explore the proteins that mediate OA- or EPA-induced insulin sensitization, a SILAC-based quantitative proteomic approach was employed.

**Figure 2:**
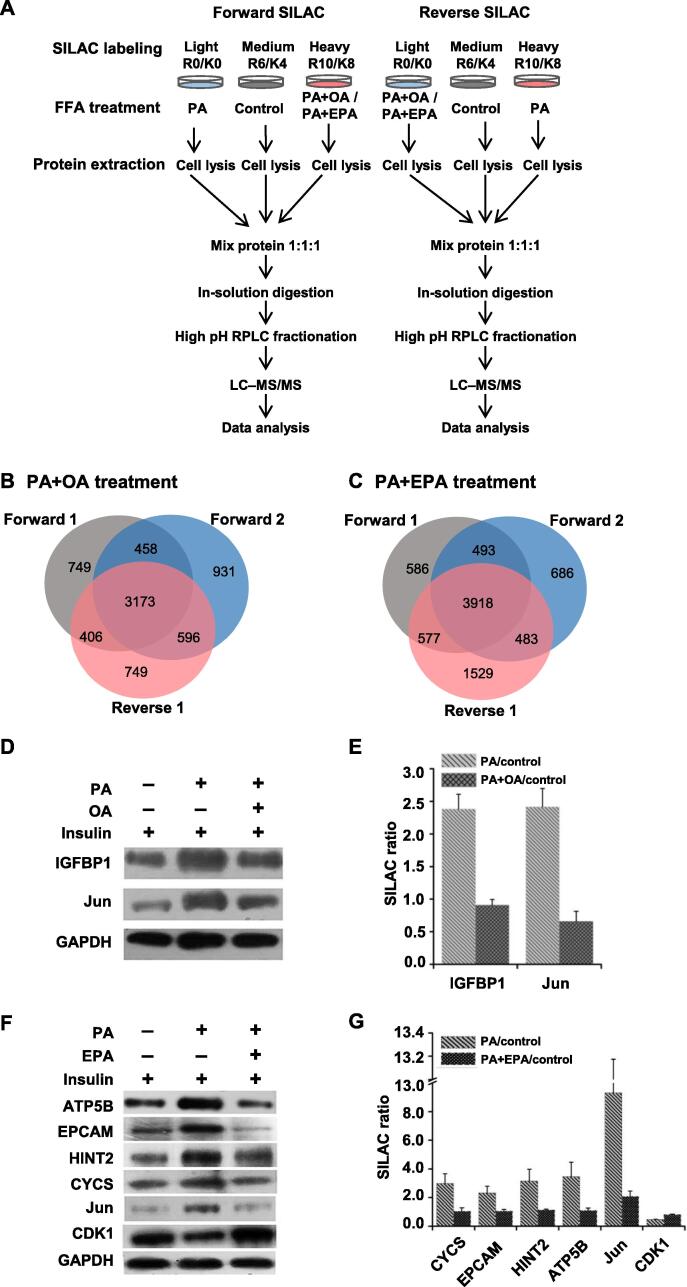
**SILAC-based quantitative proteomic analysis** **A.** Flow diagram of SILAC labeling combined with LC–MS/MS. **B.** and **C.** Quantitation overlap of the triplicate SILAC-based quantitative proteomic experiments upon PA+OA treatment (B) or PA+EPA treatment (C). Forward 1 and Forward 2 represent forward SILAC labeling experiments; Reverse 1 represents reverse SILAC labeling experiment. **D.** Western blots showing the expression of Jun and IGFBP1 in HepG2 cells treated with PA+OA treatment. **E.** SILAC quantitative proteomic ratios of Jun and IGFBP1 in HepG2 cells treated with PA+OA. **F.** Western blots showing the expression of six proteins in HepG2 cells treated with PA+EPA. **G.** SILAC quantitative proteomic ratios of six proteins in HepG2 cells treated with PA+EPA. SILAC, stable isotope labeling with amino acids in cell culture; RPLC, reversed-phase liquid chromatography; LC–MS/MS, liquid chromatography–tandem mass spectrometry; IGFBP1, insulin-like growth factor-binding protein 1; ATP5B, mitochondrial ATP synthase subunit β; EPCAM, epithelial cell adhesion molecule; HINT2, histidine triad nucleotide-binding protein 2; CYCS, cytochrome c; CDK1, cyclin-dependent kinase 1.

SILAC labeling experiments were performed in triplicate, including two forward labeling experiments and one reverse labeling experiment (Figure 2A). Further liquid chromatography–tandem mass spectrometry (LC–MS/MS) analyses quantified 4786, 5158, and 4924 proteins in HepG2 cells under PA+OA treatment (Figure 2B), and 5574, 5580, and 6507 proteins in HepG2 cells under PA+EPA treatment (Figure 2C). Only proteins that were quantified at least twice in the three replicates were used for further statistical and functional analyses (4633 proteins under PA+OA treatment and 5471 proteins under PA+EPA treatment). For these proteins, the ratios of PA/control and PA+OA/control (mean ± SD) were 1.11 ± 0.16 and 1.08 ± 0.14, respectively, while the ratios of PA/control and PA+EPA/control were 1.45 ± 0.33 and 1.06 ± 0.15, respectively. Thus, a two-fold change was considered as the threshold for significant alteration of protein expression in our analyses [18], as was shown in volcano plots of PA *versus* control, PA+OA *versus* control, and PA+EPA *versus* control, respectively (Figure S2).

To validate the accuracy of our quantification, Western blots were performed to test the expression levels of two proteins in HepG2 cells treated with PA+OA, including Jun encoded by *JUN* and insulin-like growth factor-binding protein 1 (IGFBP1), and the expression levels of six proteins in HepG2 cells treated with PA+EPA, including the mitochondrial ATP synthase subunit β (ATP5B), epithelial cell adhesion molecule (EPCAM), histidine triad nucleotide-binding protein 2 (HINT2), cytochrome c (CYCS), Jun, and cyclin-dependent kinase 1 (CDK1). The expression levels of these proteins were consistent with the ratios determined by SILAC (Figure 2D**–**G).

### Pattern analysis of SILAC results

To elucidate the influence of PA, OA, and EPA on the protein expression levels in the HepG2 cells, pattern analysis was performed as described previously [18]. Briefly, according to different responses to PA treatment, proteins were first sorted as follows: down-regulation (PA/control ≤ 0.5), no change (0.5 < PA/control < 2), and up-regulation (PA/control ≥ 2). Then, each protein was further grouped based on its response to treatment with PA+OA or PA+EPA.

In HepG2 cells treated with PA+OA, eight expression patterns (P1–P8) were defined (Table 1, Table S1). 94.9% of quantified proteins belonged to P1, which was defined as the group of proteins whose expression levels were not changed by FFA treatments. P2, P3, P5, and P7 were the main expression patterns. The proteins in P2 and P3 only responded to the PA+OA treatment. In P5 and P7, the expression levels of proteins were increased and decreased upon PA and PA+OA treatments, respectively.

**Table 1:** **Eight protein expression patterns for PA+OA treatment based on their diverse responses to FFA treatments**

	**0.5 < PA/control < 2**			**PA/control ≥ 2**			**PA/control ≤ 0.5**	
Pattern	P1	P2	P3	P4	P5	P6	P7	P8
							
No. of proteins	4399	40	15	2	143	8	18	8

*Note*: Black refers to control; red refers to PA treatment; blue refers to PA+OA treatment. PA, palmitic acid; OA, oleic acid; FFA, free fatty acid; P1–P8, Pattern 1 to Pattern 8.

In HepG2 cells treated with PA+EPA, nine expression patterns were defined (Table 2, Table S2). Similar to the results for PA+OA treatment, the majority of quantified proteins belonged to P1, in which protein expression levels were not changed by FFA treatments. The proportion of P1 (58.5%), however, was lower than that for PA+OA treatment (94.9%). The major response patterns were P2, P5, P6, P7, and P8. In P6 and P8, PA+EPA treatment resulted in a reversal of the effect of PA treatment. However, the numbers of proteins in P6 (913 proteins) and P8 (413 proteins) for PA+EPA treatment were much higher than those in P6 (8 proteins) and P8 (8 proteins) for PA+OA treatment. A similar phenomenon has also been reported in myotubes, in which the number of genes regulated by EPA treatment was larger than that regulated by OA treatment [19]. This may be due to the different effects of OA and EPA in insulin-targeted cells. Previous researchers have found that OA, but not EPA, was a more effective substrate for phospholipid synthesis in HepG2 cells [20], while EPA, but not OA, could stimulate β-oxidation in adipocytes [21].

**Table 2:** **Nine protein expression patterns for PA+EPA treatment based on their diverse responses to FFA treatments**

	**0.5 < PA/control < 2**			**PA/control ≥ 2**			**PA/control ≤ 0.5**		
Pattern	P1	P2	P3	P4	P5	P6	P7	P8	P9
								
No. of proteins	3200	282	86	6	379	913	191	413	1

*Note*: Black refers to control; red refers to PA treatment; blue refers to PA+EPA treatment. EPA, eicosapentaenoic acid; P1–P9, Pattern 1 to Pattern 9.

Since OA and EPA reversed PA-induced IR in HepG2 cells, more attention was paid to P4, P6, and P8, where PA+OA treatment or PA+EPA treatment enhanced or reversed the effect of PA treatment. Previous reports have shown that some of these proteins are related to FFA treatment, IR, and type 2 diabetes mellitus (T2DM). For example, in P4 for PA+OA treatment, cytidine deaminase (CDA) and glogin subfamily A member 7 (GOLGA7) have been reported to participate in neutrophil degranulation, which is regulated not only by PA but also by OA [22], [23]. In P6, the expression of endothelial cell selective adhesion molecule (ESAM) was up-regulated upon PA treatment, which was consistent with the previous observation that ESAM showed higher protein expression in T2DM patients with increased oxidative stress [24]. IGFBP1, which has been reported as a specific biomarker of hepatic IR [25], was also distributed to P6. The E2 ubiquitin-conjugating enzyme C (UBE2C), which participates in proteasomal degradation, was found in P8. Proteasome degradation has been reported to regulate the occurrence of hepatic IR [26]. For PA+EPA treatment, solute carrier family 27 member 1 (SLC27A1), which is not only a transporter of saturated long-chain fatty acids, such as PA [27], but also displays the ability to transport ω-3 PUFAs, was found in P4 [28]. In addition, for solute carrier family 7 member 11 (SLC7A11), which promotes cystine uptake and glutathione biosynthesis and results in the protection against oxidative stress, its expression was up-regulated not only by PA treatment in pancreatic beta-cells [29] but also by ω-3 PUFAs in macrophages [30]. Additionally, alpha/beta hydrolase domain-containing protein 4 (ABHD4), a brain N-acyl phosphatidylethanolamine (NAPE) lipase [31] that can hydrolyze various substrates with saturated, monounsaturated, or polyunsaturated N-acyl chain [32], was found in P4. Previous reports have shown that microsomal glutathione S-transferase 1 (MGST1) and microsomal glutathione S-transferase 2 (MGST2) participate in glutathione metabolism, which are closely associated with impaired insulin sensitivity in the adipocytes of obese mice [33]. MGST1 and MGST2 were present in P6. The expressive suppression of the autophagy-related protein 7 (hAGP7), which was found in P8, has also been reported to result in defective insulin signaling in the liver [34]. In all, these datasets may be helpful for dissecting the detailed regulatory mechanism of FFA-governed insulin signaling pathway.

### Signaling pathway analysis of SILAC results

**Figure 3:**
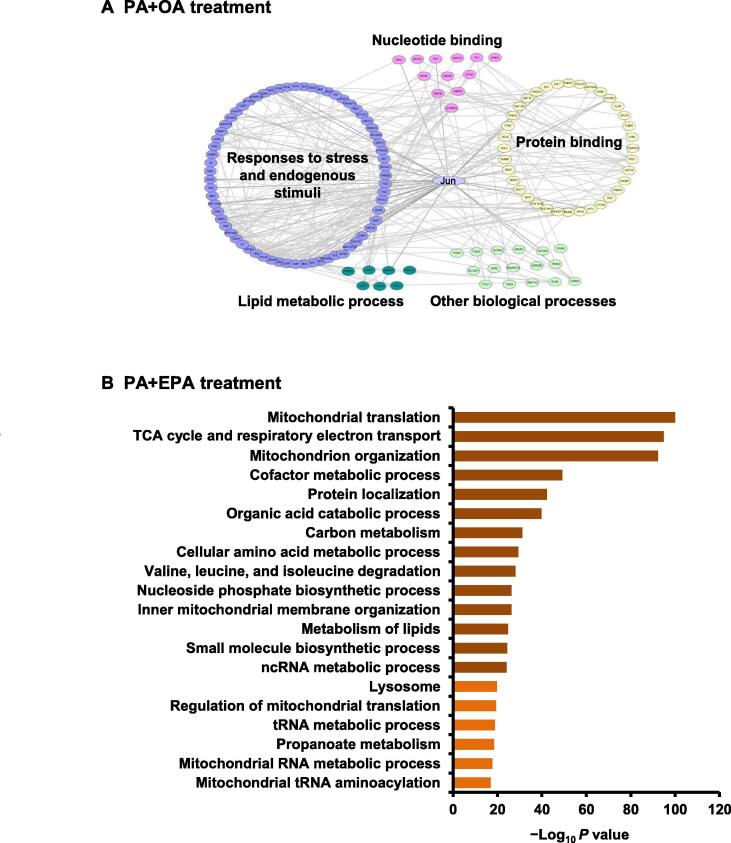
**Protein expression pattern analysis and bioinformatics analysis of quantified proteins** **A.** Network diagram of PPIs of DEPs identified in HepG2 cells treated with PA+OA. Interaction groups include responses to stress and endogenous stimuli (blue), protein binding (yellow), lipid metabolic process (dark green), nucleotide binding (pink), and other biological processes (light green). Jun is shown in light blue. **B.** Pathway enrichment analysis of DEPs identified in HepG2 cells treated with PA+EPA. The top 20 GO terms are shown. PPI, protein–protein interaction; DEP, differentially expressed protein; TCA, tricarboxylic acid.

To thoroughly understand the effects of PA and OA on cellular signaling network pathways, protein–protein interactions (PPIs) among all of the differentially expressed proteins (DEPs) were explored through the STRING database [35] and BiNGO [36]. We discovered that the most overrepresented functions of these DEPs were involved in responses to stress and endogenous stimuli, lipid metabolic process, protein binding, nucleotide binding, and other biological processes (Figure 3A).

Proteins belonging to the functional group of “responses to stress and endogenous stimuli” accounted for a relatively high proportion of all DEPs, implying that PA+OA treatment might reverse the effect of PA through this group. Proteins in the group of lipid metabolic process, such as 3-hydroxy-3-methylglutaryl-coenzyme A (HMG-CoA) reductase and microsomal triglyceride transfer protein (MTP) large subunit, seemed logical choices of proteins to guide the application of FFAs and would help us to understand the mechanism of lipid-induced IR. MTP large subunit has been shown to catalyze the transport of triglyceride and cholesteryl ester, and the inhibition of its expression significantly improved insulin sensitivity [37]. Other interaction subgroups also suggested an underlying association between FFA treatment and diverse biological functions.

To determine potential key proteins for the PA+OA treatment, we combined the results of the aforementioned pattern analysis and signaling pathway analysis. We found that the expression of Jun was up-regulated by PA treatment and reversed by PA+OA treatment (Figure 2D and E). Additionally, Jun had significantly direct and indirect interactions with proteins that participated in responses to stress and endogenous stimuli, protein binding, nucleotide binding, and lipid metabolic process (Figure 3A). Therefore, we hypothesized that *JUN* might play a key role in PA-induced hepatic IR. In normal conditions, the Jun combines with the proto-oncogene c-Fos to form the early response transcription factor complex Activator protein-1 (AP-1), which responds to diverse extracellular stimuli [38].

For DEPs in response to PA+EPA treatment, we performed Gene Ontology (GO) enrichment analysis using Metascape to understand the effects of PA and EPA on signaling network pathways and explore the potential mechanism of FFA regulation of the hepatic IR [39]. As shown in Figure 3B, the majority of DEPs were involved in mitochondria-related functions, including mitochondrial translation, the tricarboxylic acid (TCA) cycle and respiratory electron transport, mitochondrion organization, inner mitochondrial membrane organization, regulation of mitochondrial translation, mitochondrial RNA metabolic process, and mitochondrial tRNA aminoacylation. Other enriched signaling pathways and processes, including cofactor metabolic process [40], organic acid catabolic process [41], carbon metabolism [42], valine, leucine, and isoleucine degradation [43], metabolism of lipids [44], ncRNA metabolic process [45], and propanoate metabolism [46], also displayed obvious relationships with mitochondrial functions. All of these results indicate that mitochondria are important for EPA-mediated reversal of PA-induced IR, which is consistent with the previous observation that mitochondrial dysfunction is a potential mechanism contributing to fatty acid-induced hepatic IR [47].

Since EPA reversed PA-induced IR in HepG2 cells, additional GO analyses for proteins in groups P6 and P8 were performed using Metascape to further understand the PA+EPA-regulated signaling pathways. This helped us construct an intact signaling network related to FFA-mediated hepatic IR. Mitochondrial translation, the TCA cycle and respiratory electron transport, and mitochondrial protein import were the top 3 GO terms enriched in P6 (Figure S3A), indicating that mitochondria play an important role in the process of EPA-mediated reversal of PA-induced hepatic IR. In vertebrates, gluconeogenesis has been found to be the main mechanism used to regulate blood glucose level, and it contributes to approximately half of the total hepatic glucose production following overnight fasting [2]. Pyruvate carboxylase (PC), glucose-6-phosphatase 3 (G-6-Pase 3), and phosphoenolpyruvate carboxykinase (PEPCK-M), three key enzymes involved in gluconeogenesis, were also found in P6. These results were consistent with the previous observation that gluconeogenic flux could be rapidly inhibited by high level of insulin in normal liver but could be enhanced in hepatic IR [48], [49].

Dephosphorylation, ubiquitin-dependent protein catabolic process, and DNA replication were the top 3 GO terms enriched in P8 (Figure S3B). Genetic regulation of mitochondrial DNA has been reported to be closely associated with IR in obese humans [50]. A previous study has also indicated that IR accelerates muscle protein degradation through the ubiquitin–proteasome pathway [51]. Our proteomic data imply that the degradation process via the ubiquitin–proteasome pathway also occurs in the liver. Other enriched terms, such as the pentose phosphate pathway, also suggest potential biological pathways whereby EPA could reverse PA-induced IR.

### Inhibition and knockdown of ***JUN*** reverse PA-mediated reduction of AKT phosphorylation

Based on the aforementioned bioinformatics analysis for PA+OA treatment, *JUN* was found as an important factor for OA-mediated reversal of PA-induced IR. Thus, to further resolve this function of *JUN*, curcumin, a general inhibitor of *JUN* which reduces the cellular mRNA level of *JUN* [52], and specific siRNAs targeting *JUN*, were both employed to decrease the protein expression of *JUN* and investigate its influence on the insulin signaling pathway.

**Figure 4:**
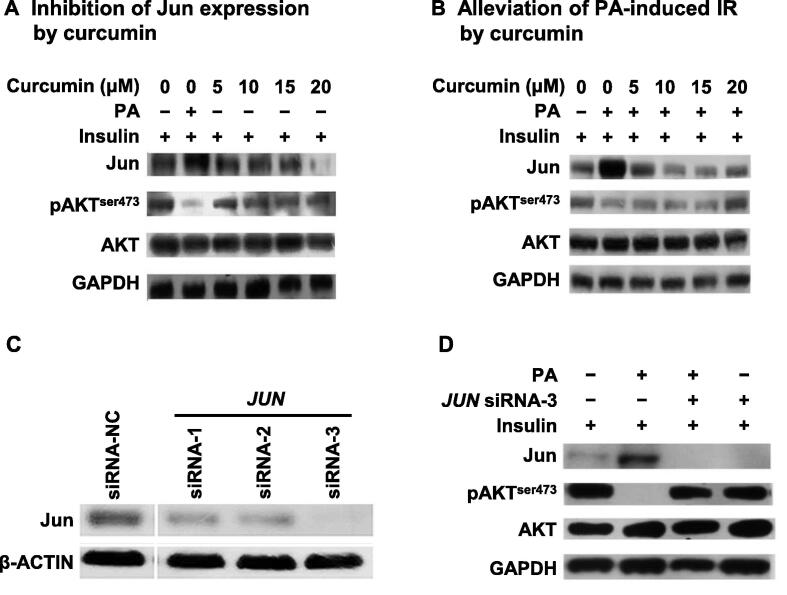
**Inhibition or knockdown of *JUN* alleviates PA-induced IR in HepG2 cells** **A.** and **B.** Curcumin inhibited the protein expression of *JUN* in a concentration-dependent pattern (A) and alleviated PA-induced IR (B). HepG2 cells were incubated with increasing concentrations of curcumin (0**–**20 μM) in the absence (A) or presence (B) of 0.5 mM PA for 12 h before insulin treatment (100 nM for 20 min at 37 °C). Western blots of total lysates were performed using antibodies for indicated proteins. **C.** Knockdown of *JUN* expression by specific siRNAs. HepG2 cells were transfected with siRNA-1, siRNA-2, and siRNA-3 that specifically targeted *JUN*. Protein expression levels of *JUN* were determined by Western blots. siRNA-NC represents a negative control siRNA. **D.** Silencing of *JUN* in HepG2 cells reversed PA-induced inhibition of AKT phosphorylation. After *JUN* was silenced, HepG2 cells were incubated in the presence and absence of 0.5 mM PA for 12 h and then treated with 100 nM insulin for 20 min. Western blots of total lysates were performed using antibodies for indicated proteins.

As shown in Figure 4A, curcumin alone inhibited the protein expression of *JUN* in a concentration-dependent manner and had no influence on the pAKT level. However, compared with PA treatment, 20 μM curcumin significantly reduced the PA-induced up-regulated protein expression of *JUN* and reversed the pAKT level. Because the protein expression of *JUN* was increased by 2-to-3 folds by PA treatment in HepG2 cells, combination treatment with curcumin and PA could not completely inhibit the protein expression of *JUN*, while curcumin alone did (Figure 4A and B). These results indicate that *JUN* might be a key mediator of PA-induced IR.

Curcumin, however, is a broad-spectrum inhibitor [53]. Therefore, a gene knockdown experiment using three *JUN*-specific siRNAs (siRNA-1/2/3) was performed. Western blots showed that all three *JUN*-specific siRNAs reduced the protein level of *JUN* in HepG2 cells with greater than 80% efficiency (Figure 4C). Since the efficiency of siRNA-3 was much higher than that of siRNA-1 and siRNA-2, it was chosen for further analysis.

As shown in Figure 4D, the pAKT level was not changed when siRNA-3 was used to silence *JUN* without PA treatment, but the inhibition of *JUN* expression with siRNA-3 could reverse the PA-reduced pAKT level. All of these results indicate that *JUN* plays a key role in PA-induced hepatic IR.

### Effects of different FFAs on intracellular adenosine triphosphate, calcium, and ROS content

Base on aforementioned pathway enrichment analysis for PA+EPA treatment, mitochondria were identified as a key factor to explain why EPA reversed PA-induced IR in HepG2 cells (Figure 3B). As an important cellular organelle, mitochondria can produce adenosine triphosphate (ATP) and small amounts of ROS to maintain microdomain cell signaling in normal conditions [54]. However, when extracellular stimulus occurs, ROS level greatly increases [54], resulting in various diseases such as cardiovascular diseases [55]. To investigate the effects of mitochondria on different FFA-regulated IR in HepG2 cells, two important functional parameters of mitochondria, the levels of ATP and ROS upon treatment with different FFAs were measured.

**Figure 5:**
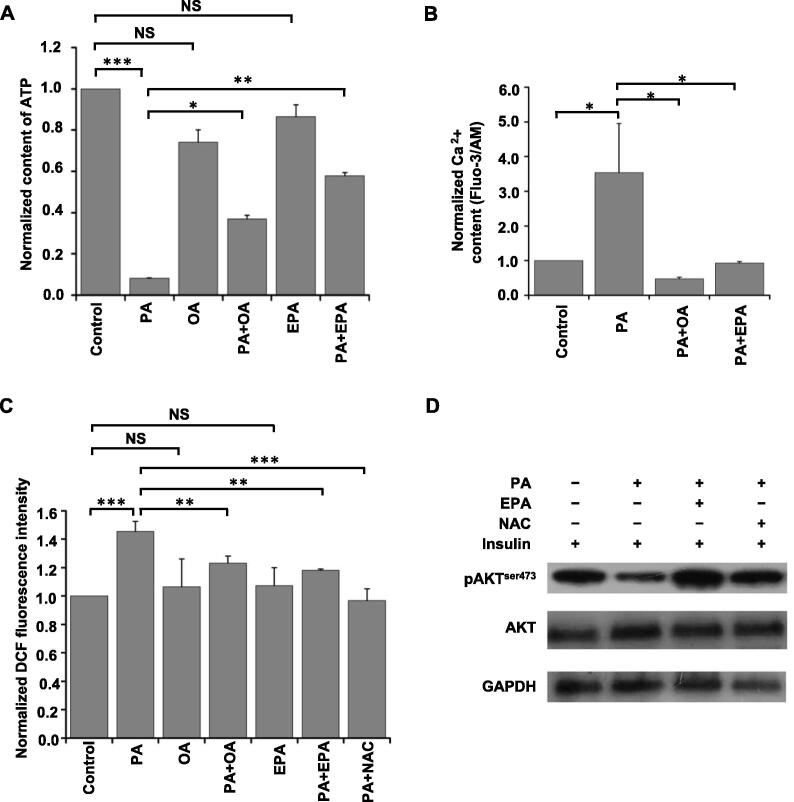
**FFAs regulate cellular ATP, Ca^2+^, and ROS content and inhibition of ROS reveres PA-induced IR in HepG2 cells** **A.** Quantification of intracellular ATP content in HepG2 cells exposed to indicated FFAs. **B.** Quantification of cystolic Ca^2+^ content in HepG2 cells exposed to indicated FFAs. **C.** Quantification of ROS content in HepG2 cells exposed to indicated FFAs with or without NAC treatment for 12 h. Data in (A–C) were normalized to the positive control (control = 1), and represented as mean ± SD from at least three independent experiments. *, *P* < 0.05; **, *P* < 0.01; ***, *P* < 0.001; NS, not significant (one-way ANOVA). **D.** Inhibiting ROS production by NAC could reverse PA-induced inhibition of pAKT. HepG2 cells were incubated with ethanol, 0.5 mM PA, 0.5 mM PA plus 0.15 mM EPA, or 0.5 mM PA plus 5 mM NAC for 12 h and then treated with 100 nM insulin for 20 min. Western blots of total lysates were performed using antibodies for indicated proteins. FFA, free fatty acid; ATP, adenosine triphosphate; ROS, reactive oxygen species; NAC, *N*-acetyl L-cysteine; DCF, dichloro fluorescein.

As shown in Figure 5A, the level of ATP was down-regulated upon PA treatment, and this down-regulation was reversed by PA+OA treatment or PA+EPA treatment. However, the extent of the reversal of ATP production by PA+OA treatment was lower than that by PA+EPA treatment. Nevertheless, EPA or OA alone did not significantly influence ATP production in HepG2 cells. Since mitochondrial Ca^2+^ overload has been reported as an important factor for decreasing ATP production in hepatocytes [56], [57], Fluo-3/AM was used to measure the Ca^2+^ content in FFA-treated HepG2 cells to determine the relationship between the intracellular Ca^2+^ content and FFA treatment. As shown in Figure 5B, PA treatment increased the Ca^2+^ content in HepG2 cells, and PA+OA treatment or PA+EPA treatment reversed this up-regulation tendency.

In contrast to the level of ATP, the level of ROS was up-regulated in PA-treated HepG2 cells, and this up-regulation was reversed by PA+EPA treatment or PA+OA treatment (Figure 5C). However, EPA or OA alone did not significantly influence ROS production. Since previous reports have shown that ROS plays an important role in the insulin signaling pathway [58], we anticipated that ROS might be a key factor in the EPA-mediated reversal of the PA-induced IR.

### Inhibition of ROS reverses PA-mediated reduction of AKT phosphorylation

To investigate the effect of ROS on PA-induced IR of HepG2 cells, *N*-acetyl L-cysteine (NAC), an effective inhibitor of ROS [59], was used to reduce the cellular production of ROS. As shown in Figure 5C, the effect of PA+NAC treatment on ROS level was similar to that of PA+OA or PA+EPA treatment. Following that, the influence of ROS on the insulin signaling pathway was investigated through monitoring the pAKT level. As shown in Figure 5D, the pAKT level was restored upon PA+NAC treatment compared to PA treatment alone. The effect of PA+NAC treatment on pAKT level was similar to that of PA+EPA treatment. These results indicate that ROS might play a crucial part in EPA-mediated reversal of PA-induced IR in HepG2 cells.

### PA, OA, and EPA all function through the ROS/*JUN* pathway in IR

As *JUN* and ROS were identified as key mediators of OA- and EPA-mediated reversal of PA-induced hepatic IR, respectively (Figure 4B, 4D, and 5D), and the protein expression patterns of *JUN* in both SILAC experiments for PA+OA and PA+EPA treatments fell into P6, we sought to investigate whether there was some connection between the protein encoded by *JUN* and ROS and whether there was a common signaling pathway for OA- and EPA-mediated reversal of IR in HepG2 cells.

**Figure 6:**
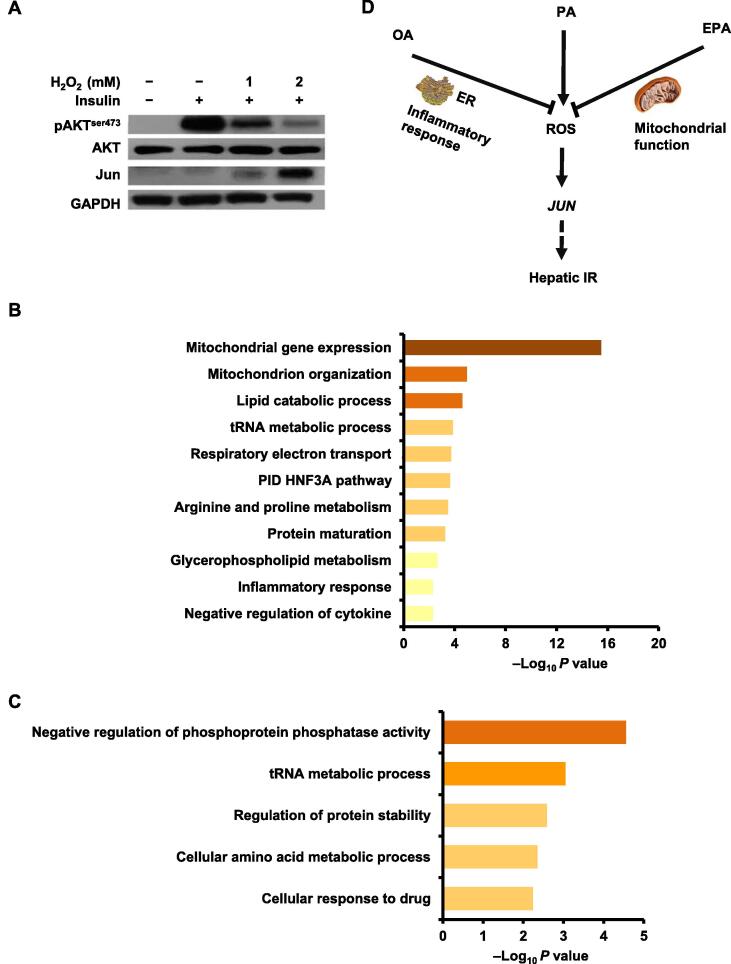
**ROS/*JUN* pathway mediates different FFA-regulated IR in HepG2 cells** **A.** H_2_O_2_ increased the protein expression of *JUN* and inhibited the insulin-stimulated AKT phosphorylation in a concentration-dependent manner. Western blots of HepG2 cells treated with ethanol, 1 mM H_2_O_2_, or 2 mM H_2_O_2_ for 12 h, respectively, before insulin treatment (100 nM for 20 min at 37 °C). Western blots of total lysates were performed using antibodies for indicated proteins. **B.** and **C.** Pathway enrichment analyses of potential *JUN*-targeted “responders” in P6 (B) and P8 (C) after FFA treatments. Representative enriched terms are shown. **D.** Schematic summarizing the effects of PA, OA, and EPA on the ROS/*JUN* pathway regulating hepatic IR. ER, endoplasmic reticulum.

As an exogenous oxidative stress, H_2_O_2_ can induce cellular generation of ROS [60], so it was applied to investigate the relationship between ROS and *JUN* and their effects on the insulin signaling pathway. As shown in Figure 6A, the protein expression of *JUN* was up-regulated in a concentration-dependent manner upon H_2_O_2_ treatment, which was reciprocally correlated to the level of pAKT. These results imply that OA, EPA, and PA regulate IR in HepG2 cells through the ROS/*JUN* pathway.

To further understand the function of *JUN* in FFA-treated HepG2 cells, the ChIP-Atlas database (https://chip-atlas.org/) [61] and the quantitative proteomic data from the PA+EPA experiment were combined. A total of 173 proteins (Table S3) were identified as potential *JUN*-targeted “responders” to FFA treatments. Among these, 65 proteins belonged to P6 and 25 proteins belonged to P8, indicating that these proteins have potential correlations with *JUN*. Further pathway enrichment analysis found that the proteins in P6 mainly functioned in mitochondria-associated processes (Figure 6B). For example, CYCS, which has been shown to play a role in oxidative phosphorylation (OXPHOS) [62], was detected as a potential *JUN*-targeted protein and displayed a similar expression tendency as Jun after FFA treatments (Figure 2F and G). All of these results indicate that *JUN*, ROS, and mitochondrial dysfunction might have a triangular relationship. However, further investigation is needed to validate this hypothesis. In addition, the *JUN*-targeted proteins in P8 mainly functioned in the negative regulation of phosphoprotein phosphatase activity and tRNA metabolic process (Figure 6C). This indicates that *JUN* has diverse functions in FFA-mediated IR in HepG2 cells.

## Discussion

The SFA PA and the MUFA OA have been reported to play opposite roles in hepatic IR, but whether the PUFA EPA also functions similarly to OA, what regulates OA- or EPA-mediated hepatic insulin sensitization, and whether they utilize the same functional mechanism, are all questions that needed to be explored. To address these questions, a SILAC-based quantitative proteomic approach and biochemical experiments were employed.

In our PA+OA experiment, we demonstrated that *JUN* was a key molecule for FFA-mediated IR in HepG2 cells. As a key member of the transcription factor AP-1, Jun plays a vital role in the inflammatory process in different tissues and diseases, such as T2DM and rheumatoid arthritis [63]. Furthermore, peroxisome proliferator-activated receptors can alleviate inflammatory responses through the negative regulation of the transcription of AP-1, which has been reported to be crucial for modulating the intersection of IR, lipid-regulated metabolic processes, and innate immune processes [64]. Therefore, we supposed that the reason that PA up-regulated while PA+OA down-regulated the protein expression of *JUN* in our results, was possibly that SFAs induced inflammation responses and MUFAs alleviated inflammation responses.

In addition to *JUN*, we also quantified many other inflammatory factors, whose PA-induced expression changes were reversed by PA+OA treatment. For example, complement *C5*, an important member of the complement system, plays a crucial role in the inflammatory system [65]. IGFBP1, which is released by the liver, has been shown to participate in the acute-phase response [66], and a previous report has already shown that IGFBP1 in plasma functions as a specific biomarker for hepatic insulin sensitivity [25]. Heme oxygenase 1 (HMOX1), which has immunomodulatory and anti-inflammatory properties, is the rate-limiting enzyme for heme degradation. The heme oxygenase (HO) system has been reported to potentiate the insulin signaling through its ability to suppress inflammatory responses [67]. Taken together, PA+OA treatment could reverse PA-induced IR through alleviating inflammation responses in HepG2 cells.

Similar to the PA+OA treatment, the PA+EPA treatment also showed a reversal effect on PA-induced IR (Figure 1F), and a quantitative proteomic technique was again employed to explore this molecular mechanism (Figure 2A). To our knowledge, this is the first proteomic study to identify potential candidates for EPA-mediated reversal of PA-induced IR in HepG2 cells. In our results, we quantified most of the functional proteins in the OXPHOS and TCA cycle, and their expression was increased upon PA treatment and reversed by PA+EPA treatment. Since the TCA cycle and respiratory electron transport are the bases and preconditions of ATP generation, the up-regulation of TCA cycle- and respiratory electron transport-related proteins could result in increase of the cellular level of ATP. However, in our data, the level of ATP was down-regulated upon PA treatment (Figure 5A), which might be due to the uncoupling effect of PA and PA-induced Ca^2+^ overload of mitochondria. The site of PA-mediated uncoupling action mainly lay in the ATPase complex, and PA could discharge the Fo membrane-associated complex to decrease ATP production [68].

Additionally, a previous report has shown that mitochondrial Ca^2+^ overload is the main reason for decreased production of ATP [57]. This was not only confirmed in our proteomic results but also in our biochemical results. The expression of mitochondrial calcium uniporter regulator 1 (MCUR1) and mitochondrial calcium uniporter (MCU), both of which can accumulate Ca^2+^ in the mitochondria [69], [70], and the cellular concentration of Ca^2+^ were up-regulated by PA treatment and were reversed by PA+EPA treatment (Figure 5B). This was the first time that PA+EPA treatment was reported to have a reversal effect on the PA-induced changes of intracellular Ca^2+^ content. In addition to the PA+EPA treatment, the PA+OA treatment could also reverse PA-induced up-regulation of Ca^2+^ content (Figure 5B). However, interestingly, PA+OA treatment did not reverse the level of ATP production as PA+EPA treatment did. The reason may be the uncoupling effect of OA on the ATPase complex to decrease the cellular production of ATP [68].

In the PA+EPA experiment, ROS was found to be a key molecule in EPA-mediated reversal of PA-induced hepatic IR. This result was not only consistent with the previous report that PA-induced excess production of ROS caused IR in adipocytes [71], but also consistent with our proteomic data in which mitochondrial proteins were grouped into the P6 expression pattern. In addition to functioning similarly to EPA in the PA-induced IR, OA has also been shown to alleviate PA-induced ROS generation (Figure 5C). However, compared with the PA+EPA experiment, mitochondrial proteins were detected in P5 of the PA+OA experiment. In P5, the expression of proteins was increased by PA treatment, but this up-regulation could not be reversed by PA+OA treatment. For example, the expression of solute carrier family 25 member 19 (SLC25A19), which mediates the uptake of thiamine pyrophosphate into the mitochondria, was reversed by PA+EPA treatment, but not by PA+OA treatment. These results implied that PA+OA treatment could not completely reverse mitochondrial dysfunction as PA+EPA treatment did. However, the reason why the level of ROS could also be reversed by PA+OA treatment remains unknown. There are some possible explanations. ROS is mainly produced by ER [72] and mitochondria [73]. ROS from ER stress is an important mediator of inflammatory responses [74], and ER stress has been reported as a potential therapeutic target for IR in pancreatic β-cells [75]. We therefore supposed that OA treatment ameliorated PA-induced inflammatory responses to decrease the cellular production of ROS from ER while EPA treatment alleviated PA-induced mitochondrial dysfunction to restore the cellular generation of ROS. However, possibly because of the tissue-specific functions of EPA, previous reports have found that EPA could not change mitochondrial functions in muscle cells [76].

Our further results revealed that ROS was an effective molecule for increasing the protein expression of *JUN*, and the increased ROS generation and protein expression of *JUN* significantly inhibited the insulin signaling pathway (Figure 6A). Previous studies have revealed that both *JUN* and ROS play important roles in inflammatory disorders [77], [78], and inflammation and oxidative stress use inflammatory cytokines as mediators to cause IR of skeletal muscle [79]. Therefore, *JUN* may be a key mediator for inflammation, ROS, and IR in HepG2 cells, and the ROS/*JUN* pathway may be an important pathway for inflammation-mediated IR and OA- and EPA-mediated reversal of PA-induced IR.

In summary, through a SILAC-based quantitative proteomic approach, we found that the ROS/*JUN* pathway was a common pathway whereby different FFAs regulated IR in HepG2 cells. Our proteomic data indicated that OA and EPA might reverse the ROS/*JUN* pathway through different mechanisms. OA regulated this pathway through preventing the cellular production of ROS from ER and ER-stress-induced inflammation response, while EPA alleviated PA-induced mitochondrial dysfunction to reduce the cellular production of ROS (Figure 6D). Additionally, we also presented many potential *JUN*-targeted candidates that might mediate the effects of FFAs on HepG2 cells. The main unanswered questions raised by this finding are how ROS regulates the protein expression of *JUN*, and by what mechanism *JUN* mediates IR. Therefore, further investigation into the underlying mechanism of the ROS- and *JUN*-mediated signaling pathways would provide important clues for understanding the molecular mechanism of FFA-mediated IR in hepatic cells.

## Materials and methods

### Cell culture

The HepG2 cell line and L02 cell line were gifts from Prof. Pengyuan Yang from the Institute of Biophysics, Chinese Academy of Sciences. HepG2 cells and L02 cells were respectively cultured in DMEM media (Catalog No. CM15019, Macgene Biotech, Beijing, China) and RPMI-1640 media (Catalog No. SH30809.01, Hyclone, Logan, UT) supplemented with 100 mg/ml streptomycin, 100 U/ml penicillin, and 10% fetal bovine serum (Catalog No. ST30-3302, FBS premium, PAN-Biotech, Adenbach, Bavaria, Germany) at 37 °C and 5% CO_2_.

### Fatty acid preparation and treatment

PA (Catalog No. P9767, Sigma-Aldrich, Taufkirchen, Germany) and OA (Catalog No. O7501, Sigma-Aldrich) were prepared according to previous methods [18]. Briefly, both FFAs were added to ethanol, making a final concentration of 80 mM and then sonicated (200 W, 4 s on bursts, and 6 s interval) by a Scientz-IID sonicator (Scientz Biotechnology, Ningbo, China) on ice until the mixture formed an emulsion. EPA (Catalog No. E6627, Sigma-Aldrich) was dissolved in ethanol to a concentration of 66 mM. Before use, different FFA stock solutions were dissolved in DMEM or RPMI-1640 complete media at 60 °C to yield concentrations as indicated. After cooling to 37 °C, these media were used to incubate HepG2 cells or L02 cells for 12 h.

### MTT assay

The toxicity of different FFAs in HepG2 cells was determined by MTT assays as described previously [80]. All of the experiments were repeated three times, and triplicate samples were measured in each experiment.

Briefly, HepG2 cells were seeded at 5000 cells per well in 96-well plates. After being cultured for 12 h, the HepG2 cells were treated with different concentrations of FFAs as indicated for another 12 h. Following that, 20 μl of sterile filtered MTT (5.0 mg/ml; Catalog No. 0793, Amresco, Solon, OH) was added to each well for 4 h. Then 150 μl of DMSO was applied to dissolve insoluble formazan crystals after any unreacted dye was removed. The absorbance at 590 nm was measured with a PerkinElmer EnSpire multimode reader (PerkinElmer, Boston, MA).

### Measurement of the effects of different FFAs on hepatic IR by Western blots

To dissect the effects of different FFAs on hepatic IR, insulin-stimulated AKT phosphorylation in hepatocytes was examined. All of the experiments were performed in triplicate. HepG2 cells were respectively treated with different concentrations of PA as indicated for 12 h, with 0.5 mM PA for different time durations as indicated, or with different concentrations of OA or EPA in the absence or presence of 0.5 mM PA for 12 h. L02 cells were also cultured in media containing either vehicle ethanol, 0.5 mM PA, 0.5 mM PA plus 0.2 mM OA, or 0.5 mM PA plus 0.15 mM EPA for 12 h to further validate the effects of OA and EPA on IR of hepatocytes.

After different FFA treatments, cells were treated with 100 nM insulin (Catalog No. P3376, Beyotime, Shanghai, China) for 20 min and then harvested for protein extraction using lysis buffer containing 8 M urea, 100 mM Tris-HCl at pH 8.5, and protease inhibitor cocktail (Catalog No. 04693132001, Roche, Basel, Switzerland) [81]. Protein concentration was measured with a Bradford assay (Catalog No. 5000205, Bio-Rad, Hercules, CA). The levels of pAKT (pAKT^Ser473^) and AKT were determined by Western blots.

For Western blots, 20 μg of protein from each sample was resolved by SDS-PAGE and then electro-transferred to PVDF membranes. PVDF membranes were then blocked and probed with phospho-AKT (pAKT^Ser473^) (1:2500; Catalog No. 4060, Cell Signaling Technology, Danvers, MA) or AKT (1:1000; Catalog No. 9272, Cell Signaling Technology) antibody. After being probed with the indicated secondary HRP antibodies [1:1000; Catalog Nos. KC-RB-035 (goat anti-rabbit IgG) and KC-MM-035 (goat anti-mouse IgG), KangCheng Bio-Tech, Shanghai, China], immunoreactive proteins on the membranes were detected by Super ECL Plus Kit (Catalog No. P1050, Applygen, Beijing, China). GAPDH (1:5000; Catalog No. KC-5G5, KangCheng Bio-Tech) was used as a loading control.

### Measurement of cellular PA content

PA was extracted according to a previous report [82]. In brief, HepG2 cells were incubated with ethanol, 0.5 mM PA, 0.5 mM PA plus 0.15 mM EPA, or 0.5 mM PA plus 0.2 mM OA for 12 h. Then, FFA-treated HepG2 cells were collected in lysis buffer after treatment with 100 nM insulin for 20 min. 90 μg of protein from each sample was added to 0.4 M NaOH/CH_3_OH and placed at room temperature for 10 min. Then 2 ml of hexane was added, and samples were mixed by vortex, followed by incubation at room temperature for 10 min. After that, the hexane phase was removed and 2 ml of 5% H_2_SO_4_/CH_3_OH was added. The mixture was kept at 70 °C for 30 min to complete the methylation of PA. Following this, 2 ml of hexane was used to extract palmitate methyl esters twice. The samples were then dried under N_2_ gas. 60 μl of hexane was used to dissolve the palmitate methyl esters for further analysis. Palmitate methyl esters were detected by GC–MS/MS using an Agilent 7890A gas chromatograph coupled with an Agilent 7000B QQQ mass spectrometer (Agilent, Santa Clara, CA). 1 μl of sample was loaded with helium as the carrier gas onto an HP-FFAP chromatographic column (30 m × 0.25 mm inner diameter, film thickness 0.25 μm; Agilent) at a flow rate of 1.0 ml/min. The injector temperature was maintained at 220 °C in the spotless mode. The GC oven settings were as follows: initial temperature, 60 °C, hold for 1 min, then increase to 180 °C at 10 °C/min, increase again to 210 °C at 3 °C/min, increase to 220 °C at 5 °C/min, and finally hold for 15 min. The ionization mode EI (70 eV, 230 °C) and a full scan of 50–550 *m*/*z* were chosen for the MS detection. The concentrations of palmitate methyl esters were measured using an external standard method. Data were acquired and processed using MassHunter Workstation Software and the NIST database.

### SILAC labeling, FFA treatment, and protein extraction

SILAC labeling was performed as previously described [18]. To generate triple SILAC labeling states, DMEM media without arginine and lysine (customized according to Catalog No. 12100061, Invitrogen, Rockford, IL) were supplemented with L-[^12^C_6_,^14^N_2_]-lysine (Lys0) and L-[^12^C_6_,^14^N_4_]-arginine (Arg0) for “light” labeling, L-[^2^H_4_]-lysine (Catalog No. DLM-2640-PK, Cambridge Isotope Laboratories, Andover, MA) and L-[^13^C_6_]-arginine (Catalog No. CLM-2265-H-1, Cambridge Isotope Laboratories) for “medium” labeling, and L-[^13^C_6_,^15^N_2_]-lysine (Catalog No. CNLM-291-H-PK, Cambridge Isotope Laboratories) and L-[^13^C_6_,^15^N_4_]-arginine (Catalog No. CNLM-539-H-1, Cambridge Isotope Laboratories) for “heavy” labeling. HepG2 cells were cultured for at least six cell population doublings in SILAC DMEM media with 10% dialyzed fetal bovine serum (Catalog No. P30-2102, PAN Biotech, Aidenbach, Germany), 100 U/ml penicillin, and 100 mg/ml streptomycin to allow complete isotopic incorporation (> 95% labeling efficiency) before treatment with different FFAs.

After SILAC labeling, the HepG2 cells were treated with different FFAs. Briefly, for forward labeling experiments, the HepG2 cells with light amino acid labeling were treated with 0.5 mM PA, the HepG2 cells with medium amino acid labeling were treated with the vehicle control, and the HepG2 cells with heavy amino acid labeling were treated with 0.5 mM PA plus 0.2 mM OA. For reverse labeling experiments, the HepG2 cells with light amino acid labeling were treated with 0.5 mM PA plus 0.2 mM OA, the HepG2 cells with medium amino acid labeling were treated with the vehicle control, and the HepG2 cells with heavy amino acid labeling were treated with 0.5 mM PA (Figure 2A). Following treatment with different FFAs for 12 h, these differentially labeled cells were incubated with 100 nM insulin for 20 min, washed twice with phosphate-buffered saline (PBS), and then scraped into lysis buffer. After sonication and centrifugation, the supernatant was collected for measuring protein concentration by Bradford assay.

The SILAC strategy for investigating the effect of EPA on PA-induced IR was similar to the strategy for OA as described above. The only difference was that the concentration of EPA used was 0.15 mM.

### In-solution digestion of SILAC-labeled proteins

An equi-mass mixture of proteins from the three labeling conditions was reduced with 10 mM dithiothreitol (DTT) at 37 °C for 2 h and then alkylated with 20 mM iodoacetamide for 45 min in the dark at room temperature. After diluting 8 M urea to 1.5 M with 25 mM ammonium bicarbonate, proteins were digested with trypsin at 37 °C overnight. Formic acid (FA) was then added to a final concentration of 0.5% to quench the digestion, and the peptide mixture was collected for further desalting after centrifugation at 20,000 *g* for 10 min.

### Peptide fractionation with high pH reversed-phase liquid chromatography

The digested peptide mixture was desalted using an Oasis HLB column (1 cc) (Catalog No. 186000383, Waters, Milford, MA). Next, a Rigol L-3000 LC system (Rigol, Beijing, China) was used to fractionate the peptides as described previously [83]. Briefly, the peptide mixtures were dissolved in 0.1% ammonium hydroxide solution (pH 10), respectively, then injected into a Xbridge Peptide BEH C18 column (150 mm × 2.1 mm, 3.5 µm particles; Waters), fractionated with high pH buffer A [2% acetonitrile (ACN), 98% H_2_O, 0.1% NH_3_H_2_O, pH 10] and buffer B (98% ACN, 2% H_2_O, 0.1% NH_3_H_2_O, pH 10) at a gradient: 4% buffer B (0 min), 8% buffer B (5 min), 18% buffer B (35 min), 32% buffer B (62 min), 95% buffer B (63 min), 95% buffer B (68 min), 5% buffer B (69 min), and 5% buffer B (76 min). Elutes were collected every 90 s, and 40 fractions were collected. Then, these fractions were merged into 10 fractions by mixing fractions 1, 11, 21, and 31; fractions 2, 12, 22, and 32; and so on. Next, all of these fractions were dried in a vacuum concentrator and kept at −80 °C until MS analysis.

### LC–MS/MS analysis

All of the high pH reversed-phase liquid chromatography (RPLC) fractionated peptides were dissolved in 0.1% FA and then automatically injected and loaded on a trap column (3 cm × 100 μm inner diameter) that was packed in house with Reprosil-Pur C18 AQ (5 μm; Dr. Maisch GmbH, Ammerbuch-Entringen, Germany). The peptides were then separated at 300 nl/min on an analytical column (18 cm × 75 μm inner diameter) packed with Reprosil-Pur C18 AQ (3 μm; Dr. Maisch GmbH) in house. Then, nano-LC was performed on an Easy-nLC 1000 HPLC system (ThermoFisher Scientific, Rockford, IL). The mobile phases were buffer A (H_2_O/0.1% FA) and buffer B (100% ACN/0.1% FA). Depending on the samples, a 78-min gradient was used for OA-treated samples and a 135-min gradient was used for EPA-treated samples. The 78-min gradient was 5%–8% buffer B for 8 min; 8%–22% buffer B for 50 min; 22%–32% buffer B for 12 min; 32%–95% buffer B for 1 min; and 95% buffer B for 7 min. The 135-min gradient was 4%–8% buffer B for 5 min; 8%–22% buffer B for 90 min; 22%–32% buffer B for 22 min; 32%–95% buffer B for 3 min; and 95% buffer B for 15 min.

The nano-LC was connected online to a Q Exactive Hybrid Quadrupole-Orbitrap mass spectrometer (ThermoFisher Scientific), which was operated in the positive ion and data-dependent acquisition (DDA) modes. The automatic gain control (AGC) target value and maximum injection time for the full MS scan were set as 3 × 10^6^ ions and 60 ms, respectively. Each MS scan was acquired at a high resolution (70000 at *m*/*z* 200) and the mass range was 300–1600 *m*/*z*. The AGC target value and the maximum injection time for MS/MS were set as 5 × 10^4^ and 80 ms, respectively. The dynamic exclusion time was 40 s. MS/MS spectra were captured at a resolution of 17,500 at *m*/*z* 200. The 20 most abundant peptide ions were chosen for higher-energy C-trap dissociation (HCD) fragmentation if they were at least doubly charged. For the nano electrospray ion (ESI) source setting, the spray voltage was set at 2.0 kV without sheath gas flow, and the capillary temperature was 320 °C.

### MS data processing

The raw MS data were analyzed with Proteome Discoverer (version 1.4, ThermoFisher Scientific). SEQUEST HT was used for protein identification and Percolator was used for evaluation of the false discovery rate (FDR) for protein identification. The UniProt human protein database (updated on February 2016, including 92,382 entries), supplemented with 247 known contaminants, was used for the database search. The database search parameters were as follows: 1) trypsin was chosen as the digestive enzyme, and no more than two missed cleavages were allowed; 2) 10 ppm and 0.02 Da were used for the mass tolerance of precursor ions and product ions, respectively; 3) cysteine carbamidomethylation was chosen as a fixed modification; and 4) stable isotope-labeled arginine (Arg6/Arg10) and lysine (Lys4/Lys8) for SILAC and methionine oxidation were set as variable modifications. The peptide confidence parameter was set as high. A FDR < 1% was used for protein identification.

### Data analysis

For quantification of MS data, only those proteins that were quantified at least twice and with a fold change in expression more than 2 or less than 0.5 were considered as DEPs and chosen for further bioinformatics analysis. Volcano plots were performed through Microsoft Excel and R statistical software, the results of which displayed the corresponding *P* values and mean values of log_2_ ratio (PA/control) of the biological triplicates. *t*-test analyses were performed for the binary comparisons in the volcano plots.

A PPI network analysis was performed using the STRING database [35]. The PPI network was then visualized using the Cytoscape program and further analyzed with the program BiNGO [36].

Pathway enrichment analysis for DEPs was performed using Metascape [39] (https://metascape.org). All of the genes from the genome were selected as the enrichment background. The enrichment parameters were set as: 1) *P* value < 0.01, 2) minimum count of 3, and 3) enrichment factor > 1.5. The enrichment factor refers to the ratio of the observed count and the expected count by chance. DEPs with aforementioned parameters were grouped into clusters according to their membership similarities.

To further investigate the function of *JUN* in FFA-treated HepG2 cells, ChIP-Atlas (https://chip-atlas.org/) [61] was used to predict the target genes of *JUN*. Then, the potential target genes (binding scores of MACS2 and STRING > 0, distance from transcription start site = 1 kb) were combined with proteomic data to discover potential *JUN*-targeted “responders” to FFA treatments. Finally, pathway enrichment analysis was further performed using Metascape.

### Verification of SILAC-based quantitative proteomic results

To validate the OA-treated SILAC results, HepG2 cells were treated with ethanol, 0.5 mM PA, and 0.5 mM PA plus 0.2 mM OA for 12 h, respectively, and then treated with 100 nM insulin for 20 min. Proteins were then extracted, and Western blots were performed to detect the expression levels of Jun (1:1000; Catalog No. 9165T, Cell Signaling Technology) and IGFBP1 (1:1000; Catalog No. 31025T, Cell Signaling Technology).

To validate the EPA-treated SILAC results, HepG2 cells were treated with ethanol, 0.5 mM PA, and 0.5 mM PA plus 0.15 mM EPA for 12 h, respectively, and then treated with 100 nM insulin for 20 min. Proteins were then extracted, and Western blots were performed to detect the expression levels of Jun, CYCS (1:500; Catalog No. 31025T, BD Biosciences, San Jose, CA), ATP5B (1:1000; Catalog No. ab14730, Abcam, Cambridge, MA), HINT2 (1:500; Catalog No. ab100871, Abcam), EPCAM (1:1000; Catalog No. 2626, Cell Signaling Technology), and CDK1 (1:2000; Catalog No. 9116, Cell Signaling Technology).

### Inhibition of the expression of ***JUN*** with curcumin

To investigate whether the inhibition of *JUN* could affect insulin-stimulated AKT phosphorylation in HepG2 cells, curcumin (Catalog No. 08511, Sigma-Aldrich) which is a general inhibitor of *JUN*, was used to reduce the protein expression of *JUN*. Briefly, HepG2 cells were cultured in different media containing increasing concentrations of curcumin (5 μM, 10 μM, 15 μM, and 20 μM) with or without 0.5 mM PA for 12 h. After treatment, the cells were treated with 100 nM insulin for 20 min. Proteins were extracted. AKT, pAKT, and the protein expression of *JUN* and *GAPDH* were detected by Western blots.

### siRNA-mediated knockdown of ***JUN***

Three specific siRNA sequences for *JUN* and a negative control siRNA (siRNA-NC) sequence were obtained from Gene Pharma (Shanghai, China). The three specific siRNA sequences for *JUN* were: 5′-AAGAACGTGACAGATGAGCAG-3′ (siRNA1) [84], 5′-CCAAGAACGUGACAGAUGATT-3′ (siRNA2) [85], and 5′-AGAUGGAAACGACCUUCUATT-3′ (siRNA3) [86]. The siRNA-NC sequence was 5′-UUCUCCGAACGUGUCACGUTT-3′. Lipofectamine 3000 Transfection Reagent (Catalog No. L3000001, Invitrogen) was used to mediate the transfection of siRNAs. Briefly, 100 nM of specific siRNAs or siRNA-NC were incubated with Lipofectamine 3000 for 20 min, and were then diluted in Opti-MEM (Catalog No. 31985070, Life Technologies, Carlsbad, CA). Following that, freshly passaged cells (1.8 × 10^6^) and siRNA-Lipofectamine mixture were plated in six-well cell culture plates. After 6 h of incubation, the transfection medium was replaced with fresh DMEM complete medium. After 60-h transfection, proteins were extracted and the efficiency of siRNA knockdown was evaluated by Western blots. β-ACTIN (1:5000; Catalog No. HX18271, Huaxing Bioscience, Beijing, China) was used as a loading control.

After *JUN* was silenced, HepG2 cells were incubated in the presence and absence of 0.5 mM PA for 12 h and then treated with 100 nM insulin for 20 min. AKT, pAKT, and the protein expression of *JUN* were determined by Western blots.

### Measurement of cellular ATP content in FFA-treated HepG2 cells

The intracellular ATP content was determined using a luciferase/luciferin ATP detection kit (Catalog No. S0026, Beyotime). In brief, HepG2 cells were incubated with ethanol, 0.5 mM PA, 0.5 mM PA plus 0.15 mM EPA, 0.5 mM PA plus 0.2 mM OA, 0.2 mM OA, or 0.15 mM EPA for 12 h. Then, FFA-treated HepG2 cells were collected and washed with PBS after treatment with 100 nM insulin for 20 min. Next, 100 μl of lysis buffer from the ATP detection kit was added to each sample, and samples were incubated for 10 min at room temperature. Cell lysates were then collected after centrifugation for measuring ATP content. Luminescence was immediately detected using a Thermo Scientific Varioskan Flash luminometer (ThermoFisher Scientific). The relative ATP level was normalized to the number of cells.

### Determination of intracellular calcium content

The fluorescence intensity of Fluo-3/AM (Catalog No. S1056, Beyotime) was used to measure the content of Ca^2+^ [87]. In brief, HepG2 cells were incubated with ethanol, 0.5 mM PA, 0.5 mM PA plus 0.15 mM EPA, or 0.5 mM PA plus 0.2 mM OA for 12 h. After FFA treatments, the HepG2 cells were cultured with 5 µM Fluo-3/AM for 1 h at 37 °C in the dark. The Fluo-3/AM loaded cells were trypsinized, washed three times with PBS, and then resuspended with PBS. Green fluorescence from Fluo-3/AM was measured with a FACSCalibur flow cytometer (BD Biosciences). The fluorescence intensity of 20,000 labeled cells was measured for each analysis. The assay was repeated three times or more.

### Measurement of cellular ROS content

The fluorescent probe 2,7-dichlorodihydrofluorescein diacetate (DCDHF-DA) (Catalog No. C1300, Applygen, Beijing, China) was used to measure the content of intracellular ROS [88]. Briefly, HepG2 cells were incubated with ethanol, 0.5 mM PA, 0.2 mM OA, 0.5 mM PA plus 0.2 mM OA, 0.15 mM EPA, 0.5 mM PA plus 0.15 mM EPA, or 0.5 mM PA plus 5 mM NAC (Catalog No. A9165, Sigma-Aldrich). Then, the FFA-treated HepG2 cells were washed with PBS and cultured in 10 µM DCDHF-DA for 1 h at 37 °C after treatment with 100 nM insulin for 20 min. After that, cells were harvested and washed with PBS twice. The cell pellets were resuspended and analyzed for intracellular ROS with a Thermo Scientific Varioskan Flash (ThermoFisher Scientific). The assay was repeated at least three times, and each sample was assayed three times. The data were normalized to the number of cells harvested.

### Measurement of the effect of NAC on PA-induced IR

NAC, an effective inhibitor of ROS [59], was used to investigate the effect of ROS on EPA-mediated reversal of PA-induced IR. Briefly, HepG2 cells were incubated with ethanol, 0.5 mM PA, 0.5 mM PA plus 0.15 mM EPA, or 0.5 mM PA plus 5 mM NAC for 12 h. They were then treated with 100 nM insulin for 20 min. After that, proteins were extracted, and the levels of pAKT, AKT, and GAPDH were monitored by Western blots.

### Determination of the effects of H_2_O_2_ on the protein expression of ***JUN*** and IR in HepG2 cells

To investigate whether ROS takes part in the *JUN*-mediated insulin signaling pathway, H_2_O_2_, which participates in mitochondrial ROS generation [60], was used to stimulate the protein expression of *JUN*. Briefly, HepG2 cells were treated with ethanol, 1 mM H_2_O_2_, or 2 mM H_2_O_2_ for 12 h. Next, 100 nM insulin was used to stimulate the cells for 20 min. Proteins were extracted, and AKT, pAKT, and the protein expression of *JUN* and *GAPDH* were detected by Western blots.

## Data availability

All of the MS proteomics data were uploaded via the PRoteomics IDEntifications database (PRIDE: PXD012066 for the PA+OA experiment; PXD012065 for the PA+EPA experiment), which are publicly accessible at https://proteomecentral.proteomexchange.org.

## CRediT author statement

**Yaping Sun:** Conceptualization, Formal analysis, Visualization, Writing - original draft, Writing - review & editing, Methodology, Validation. **Jifeng Wang:** Methodology, Validation. **Xiaojing Guo:** Resources. **Nali Zhu:** Methodology, Validation, Funding acquisition. **Lili Niu:** Resources. **Xiang Ding:** Resources. **Zhensheng Xie:** Resources. **Xiulan Chen:** Conceptualization, Formal analysis, Visualization, Writing - original draft, Writing - review & editing. **Fuquan Yang:** Conceptualization, Formal analysis, Visualization, Writing - original draft, Writing - review & editing, Funding acquisition. All authors have read and approved the final manuscript.

## Competing interests

The authors have stated that no competing interests exist.

## Supplementary material

Supplementary Figure S1 **Effects of different FFAs on the cellular content of PA in HepG2 cells and the inhibitory effect of OA or EPA on PA-induced impairment of insulin signaling in human L02 cells**. **A**. HepG2 cells were exposed to the indicated fatty acids for 12 h. Intracellular PA content was then quantified. 90 μg of proteins per sample was used to extract PA. PA was esterified through the methyl esterification buffer (5% H_2_SO_4_/CH_3_OH), extracted by hexane, then dried under N2 gas, and dissolved in 60 μl hexane. The concentration of palmitate methyl ester was tested using the external standard method by gas chromatography-tandem mass spectrometry (GC-MS/MS). Data represent the mean ± SD from at least three independent experiments. *, *P* < 0.05; **, *P* < 0.01. Data were normalized relative to the positive control (control = 1). **B**. L02 cells were treated with vehicle ethanol, 0.5 mM PA, 0.5 mM PA plus 0.2 mM OA, or 0.5 mM PA plus 0.15 mM EPA for 12 h before insulin treatment (100 nM for 20 min at 37 °C). Western blots of total lysates were performed using antibodies for indicated proteins.

Supplementary Figure S2 **Differentially expressed proteins after FFA treatment**. **A–D**. Volcano plots of the PA/control (A for PA plus OA experiment and B for PA plus EPA experiment), PA+OA/control (C), and PA+EPA/control (D), wherein the significantly (*P* < 0.05) up-regulated (red) and down-regulated (blue) proteins were highlighted.

Supplementary Figure S3 **Functional analysis of the differentially expressed proteins in patterns P6 and P8 of PA+EPA experiment**. **A**. and **B**. Signaling process and pathway enrichment analysis of the differentially expressed proteins in pattern P6 (A) and pattern P8 (B) by Metascape suggested that diverse biological signaling pathways could participate in EPA-mediated reversal of PA-induced IR. The top 20 clusters are shown with their representative enriched terms.
