# Supplementary figures and images for: Oleic Acid and Eicosapentaenoic Acid Reverse Palmitic Acid-induced Insulin Resistance in Human HepG2 Cells via the Reactive Oxygen Species/JUN Pathway
Source: Genomics Proteomics Bioinformatics. 2021 Feb 23;19(5):754–71. doi: 10.1016/j.gpb.2019.06.005 (PMC9170756; doi:10.1016/j.gpb.2019.06.005)

## Slide 1
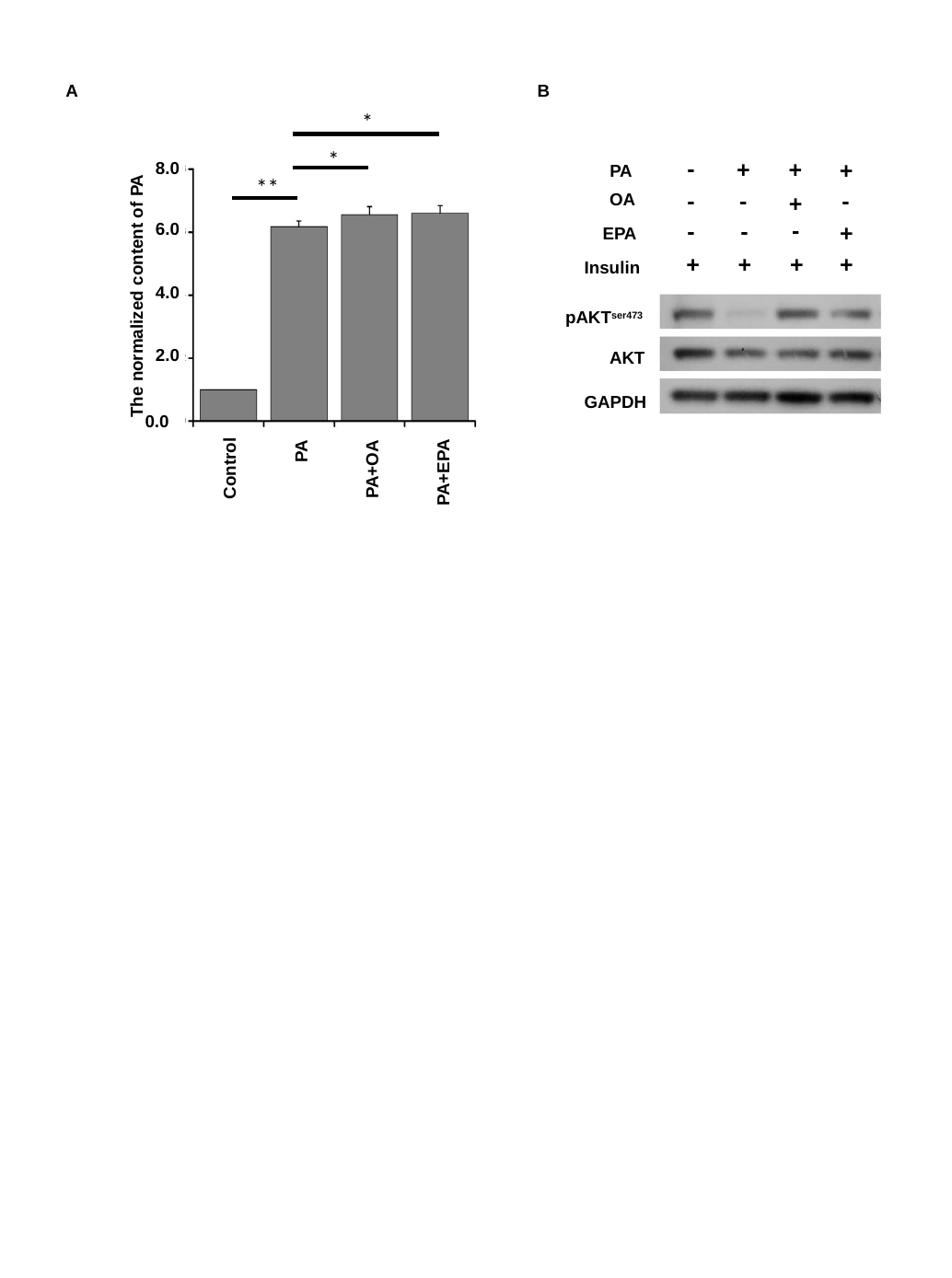

A
*
**
*
6.0
The normalized content of PA
4.0
2.0
0.0
PA
Control
PA+OA
PA+EPA
8.0
B
-
PA
 - -
+
OA
 -
EPA
 + + +
Insulin
pAKTser473
AKT
GAPDH
+ +
+
-
-
-
+
+

Supplement: Supplementary Figure S1 — Effects of different FFAs on the cellular content of PA in HepG2 cells and the inhibitory effect of OA or EPA on PA-induced impairment of insulin signaling in human L02 cells. A. HepG2 cells were exposed to the indicated fatty acids for 12 h. Intracellular PA content was then quantified. 90 μg of proteins per sample was used to extract PA. PA was esterified through the methyl esterification buffer (5% H2SO4/CH3OH), extracted by hexane, then dried under N2 gas, and dissolved in 60 μl hexane. The concentration of palmitate methyl ester was tested using the external standard method by gas chromatography-tandem mass spectrometry (GC-MS/MS). Data represent the mean ± SD from at least three independent experiments. *, P < 0.05; **, P < 0.01. Data were normalized relative to the positive control (control = 1). B. L02 cells were treated with vehicle ethanol, 0.5 mM PA, 0.5 mM PA plus 0.2 mM OA, or 0.5 mM PA plus 0.15 mM EPA for 12 h before insulin treatment (100 nM for 20 min at 37 °C). Western blots of total lysates were performed using antibodies for indicated proteins. [file mmc1.pptx]
