# Supplementary material for: Oleic Acid and Eicosapentaenoic Acid Reverse Palmitic Acid-induced Insulin Resistance in Human HepG2 Cells via the Reactive Oxygen Species/JUN Pathway
Source: Genomics Proteomics Bioinformatics. 2021 Feb 23;19(5):754–71. doi: 10.1016/j.gpb.2019.06.005 (PMC9170756; doi:10.1016/j.gpb.2019.06.005)
Supplement: Supplementary Figure S2 — Differentially expressed proteins after FFA treatment. A–D. Volcano plots of the PA/control (A for PA plus OA experiment and B for PA plus EPA experiment), PA+OA/control (C), and PA+EPA/control (D), wherein the significantly (P < 0.05) up-regulated (red) and down-regulated (blue) proteins were highlighted. [file mmc2.pptx]

## Slide 1
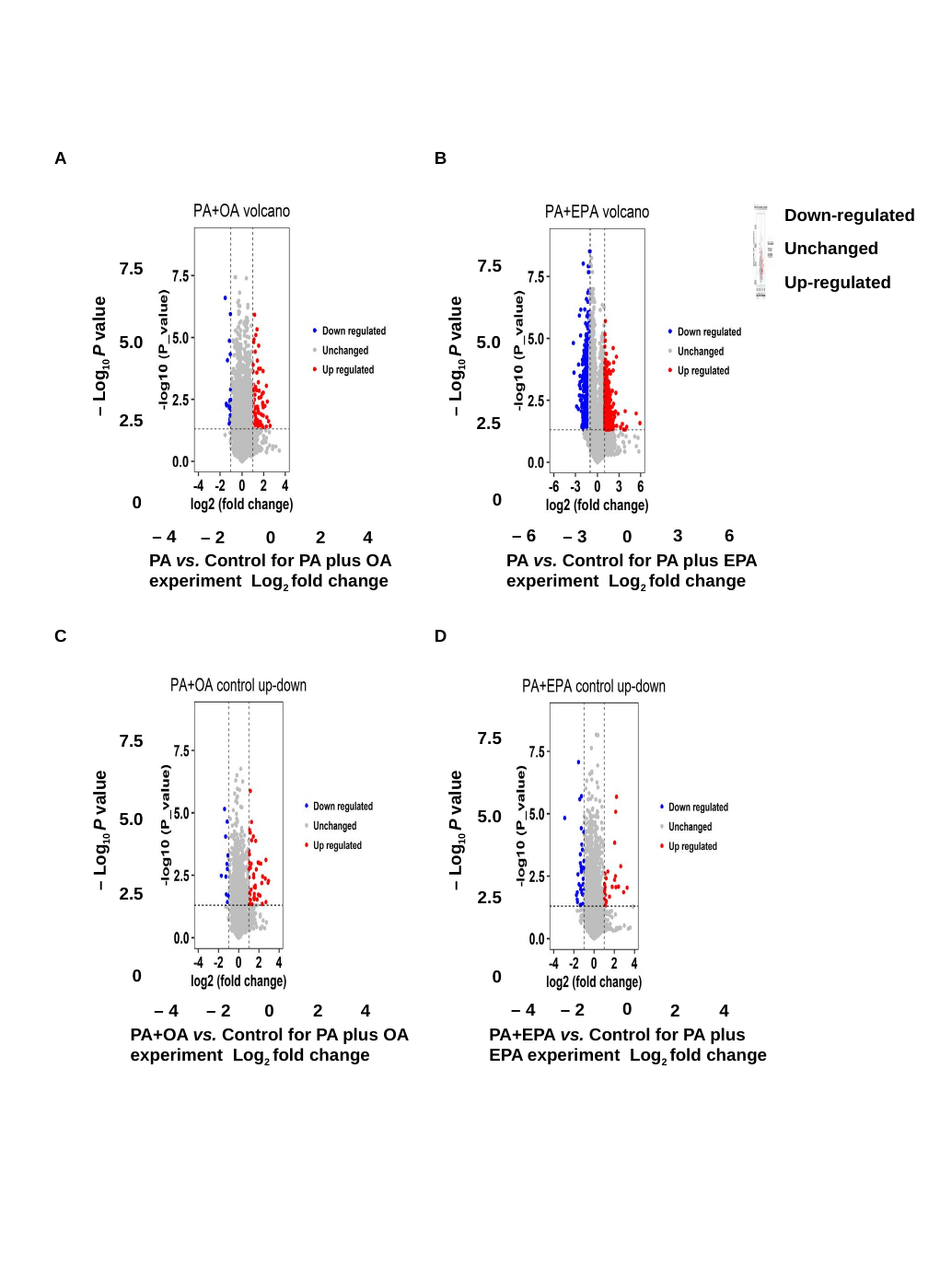

A
B
7.5
5.0
2.5
0
– 4
2
– 2
0
4
Down-regulated
Unchanged
Up-regulated
7.5
5.0
– Log10 P value
– Log10 P value
2.5
0
– 6
3
6
– 3
0
PA vs. Control for PA plus OA experiment Log2 fold change
PA vs. Control for PA plus EPA experiment Log2 fold change
C
D
7.5
5.0
2.5
0
0
– 4
– 2
2
4
7.5
5.0
– Log10 P value
– Log10 P value
2.5
0
– 4
2
– 2
0
4
PA+EPA vs. Control for PA plus EPA experiment Log2 fold change
PA+OA vs. Control for PA plus OA experiment Log2 fold change
