# Supplementary material for: Oleic Acid and Eicosapentaenoic Acid Reverse Palmitic Acid-induced Insulin Resistance in Human HepG2 Cells via the Reactive Oxygen Species/JUN Pathway
Source: Genomics Proteomics Bioinformatics. 2021 Feb 23;19(5):754–71. doi: 10.1016/j.gpb.2019.06.005 (PMC9170756; doi:10.1016/j.gpb.2019.06.005)
Supplement: Supplementary Figure S3 — Functional analysis of the differentially expressed proteins in patterns P6 and P8 of PA+EPA experiment. A. and B. Signaling process and pathway enrichment analysis of the differentially expressed proteins in pattern P6 (A) and pattern P8 (B) by Metascape suggested that diverse biological signaling pathways could participate in EPA-mediated reversal of PA-induced IR. The top 20 clusters are shown with their representative enriched terms. [file mmc3.pptx]

## Slide 1
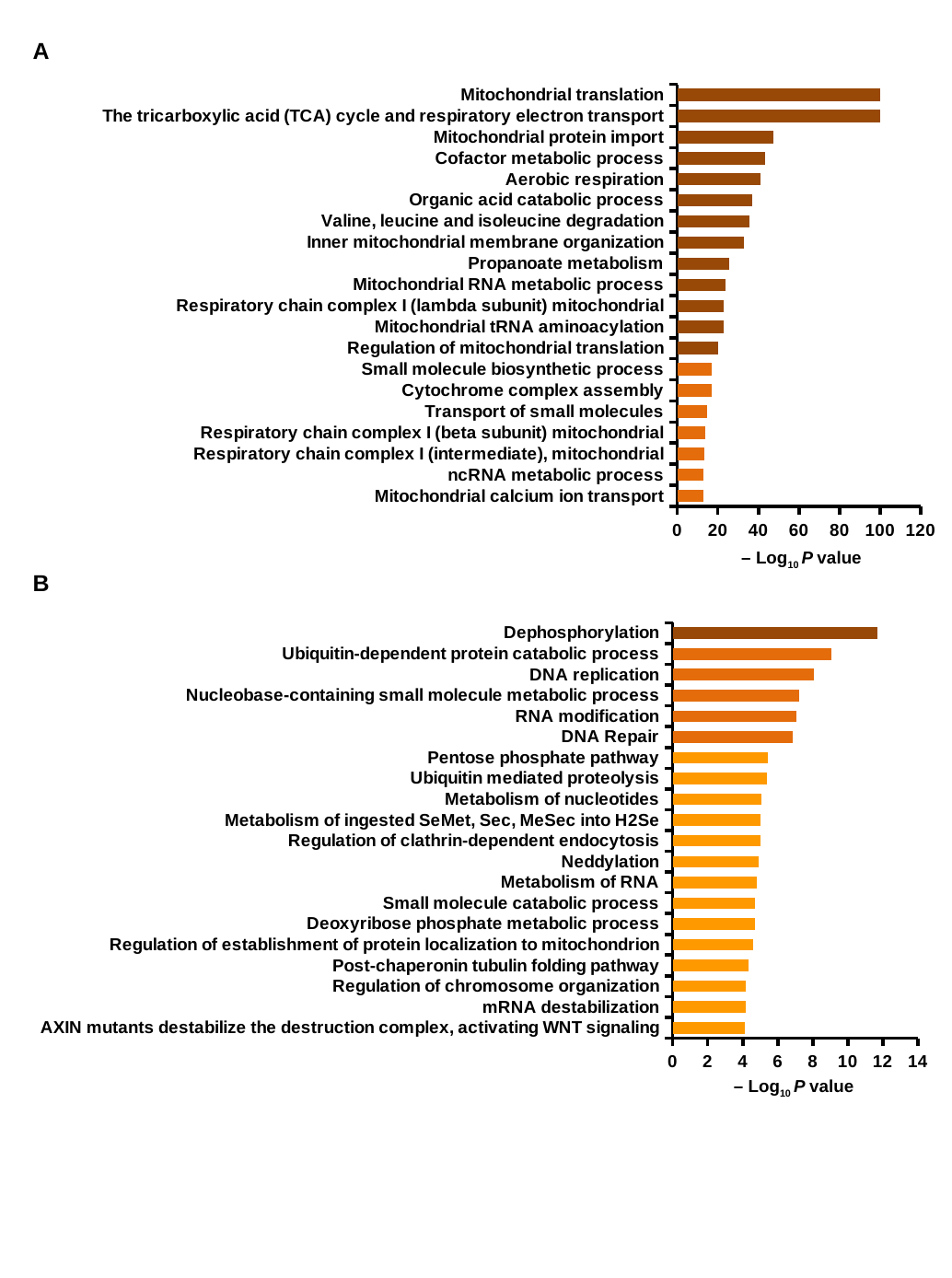

### Chart
| Category | |
|---|---|
| Mitochondrial calcium ion transport | 12.75 |
| ncRNA metabolic process | 12.75 |
| Respiratory chain complex I (intermediate), mitochondrial | 13.32 |
| Respiratory chain complex I (beta subunit) mitochondrial | 14.02 |
| Transport of small molecules | 14.67 |
| Cytochrome complex assembly | 17.07 |
| Small molecule biosynthetic process | 17.08 |
| Regulation of mitochondrial translation | 20.29 |
| Mitochondrial tRNA aminoacylation | 22.78 |
| Respiratory chain complex I (lambda subunit) mitochondrial | 22.98 |
| Mitochondrial RNA metabolic process | 23.96 |
| Propanoate metabolism | 25.76 |
| Inner mitochondrial membrane organization | 32.83 |
| Valine, leucine and isoleucine degradation | 35.72 |
| Organic acid catabolic process | 37.07 |
| Aerobic respiration | 41.18 |
| Cofactor metabolic process | 43.23 |
| Mitochondrial protein import | 47.5 |
| The tricarboxylic acid (TCA) cycle and respiratory electron transport | 100.0 |
| Mitochondrial translation | 100.0 |A
### Chart
| Category | |
|---|---|
| AXIN mutants destabilize the destruction complex, activating WNT signaling | 4.14 |
| mRNA destabilization | 4.16 |
| Regulation of chromosome organization | 4.19 |
| Post-chaperonin tubulin folding pathway | 4.32 |
| Regulation of establishment of protein localization to mitochondrion | 4.61 |
| Deoxyribose phosphate metabolic process | 4.68 |
| Small molecule catabolic process | 4.69 |
| Metabolism of RNA | 4.82 |
| Neddylation | 4.89 |
| Regulation of clathrin-dependent endocytosis | 5.0 |
| Metabolism of ingested SeMet, Sec, MeSec into H2Se | 5.01 |
| Metabolism of nucleotides | 5.05 |
| Ubiquitin mediated proteolysis | 5.38 |
| Pentose phosphate pathway | 5.44 |
| DNA Repair | 6.87 |
| RNA modification | 7.08 |
| Nucleobase-containing small molecule metabolic process | 7.24 |
| DNA replication | 8.07 |
| Ubiquitin-dependent protein catabolic process | 9.07 |
| Dephosphorylation | 11.68 |– Log10 P value
B
– Log10 P value
